# Effect of tardive dyskinesia on quality of life in patients with bipolar disorder, major depressive disorder, and schizophrenia

**DOI:** 10.1007/s11136-019-02269-8

**Published:** 2019-08-21

**Authors:** Joseph McEvoy, Sanjay K. Gandhi, Avery A. Rizio, Stephen Maher, Mark Kosinski, Jakob Bue Bjorner, Benjamin Carroll

**Affiliations:** 1 Medical College of Georgia, Augusta, GA USA; 2 Teva Pharmaceuticals Frazer, 41 Moores Rd, Malvern, PA 19355 USA; 3 Optum, Johnston, RI USA

**Keywords:** Tardive dyskinesia, Health-related quality of life, Schizophrenia, Bipolar disorder, Major depressive disorder, Patient-reported outcome

## Abstract

**Purpose:**

Tardive dyskinesia (TD) is a common but serious hyperkinetic movement disorder and side effect of antipsychotic medications used to treat bipolar disorder (BD), major depressive disorder (MDD), and schizophrenia (SZ). The purpose of this study was to evaluate health-related quality of life (HRQoL) in a population with diagnoses for BD, MDD, or SZ by comparing patients with TD (*n* = 197) with those without TD (*n* = 219). HRQoL in each group was also compared with HRQoL of the general population.

**Methods:**

This study employed a cross-sectional web-based survey. HRQoL was assessed by four instruments: the SF-12 Health Survey, Version 2 (SF-12v2), the Quality of Life Enjoyment and Satisfaction Questionnaire, Short Form (Q-LES-Q-SF), the Social Withdrawal subscale of the Internalized Stigma of Mental Illness Scale (SW-ISMI); and two questions on movement disorders.

**Results:**

Patients with TD had significantly worse HRQoL and social withdrawal than those without. The differences were more pronounced for physical HRQoL domains than for mental health domains. Patients with more-severe TD, assessed through either self-rating or clinician rating, experienced significantly worse HRQoL than did those with less-severe TD. The impact of TD was substantially greater in patients with SZ than in those with BD or MDD. Compared with the general population, patients with BD, MDD, or SZ experienced significantly worse HRQoL regardless of TD status, although this deficit in HRQoL was greater among those with TD.

**Conclusions:**

The presence of TD is associated with worse HRQoL and social withdrawal. The most severe impact of TD is on physical aspects of patients’ HRQoL.

## Introduction

Tardive dyskinesia (TD) is an often irreversible hyperkinetic movement disorder that is typically caused by exposure to antipsychotic medications used for the treatment of mental illnesses, including bipolar disorder (BD), major depressive disorder (MDD), and schizophrenia (SZ) [1–5]. Symptoms of TD are characterized by involuntary and repetitive movements that most commonly affect the face, mouth, and tongue, but can also manifest in the extremities [6, 7]. These can range from mild to severe, with involuntary movements being localized or widespread [2, 5]. The severity of TD symptoms can be assessed using clinician-reported outcome assessments such as the Abnormal Involuntary Movement Scale (AIMS) [8]; however, AIMS alone does not sufficiently capture the full impact of TD on a patient’s health-related quality of life (HRQoL).

It is difficult to rate the impact of TD on a patient’s HRQoL by rating the severity of abnormal movements alone, because even subtle involuntary movements in the facial area can have substantial negative social and emotional impacts. For those with TD, social and emotional impacts have been described as the most debilitating aspects of living with the condition [9]. For instance, involuntary movements associated with the typical oro-bucco-lingual dyskinesia such as frowning, tongue twisting or thrusting, and lip smacking and puckering can cause difficulty for the patients in fully participating in their communities or in maintaining employment [2, 10]. A recent study reported that many of the most common impacts of TD were social and emotional in nature, including unwanted social attention, feeling embarrassed, and social isolation [11].

The prevalence of TD has been estimated to increase over the coming decade [12]. This is due in part to the increased incidence of early onset schizophrenia, as well as the expanded use of antipsychotics in broader patient population [13–15]. Despite this increasing prevalence, the effect of TD on patients’ HRQoL has not been thoroughly investigated. For individuals with schizophrenia, previous studies have shown that those who have TD have higher mortality rates and poorer quality of life than those without TD; however, these studies had a relatively small sample size and included only patients with schizophrenia [16, 17]. Very little research is available on the impacts of TD in non-schizophrenia populations, such as those with BD or MDD. These research gaps indicate the need to more fully explore the relationship between TD and HRQoL, particularly among a sample of patients with respect to varying psychiatric diagnoses.

As such, the current study was undertaken to better understand the impact of TD on HRQoL and social withdrawal among individuals also diagnosed with BD, MDD, or SZ. To our knowledge, this is the first study to describe HRQoL in patients with TD across multiple psychiatric conditions.

## Methods

### Study design

This study utilized a cross-sectional web-based survey of adult psychiatric patients with clinician-confirmed diagnosis of TD or clinician-confirmed absence of TD. This study was exploratory and quasi-experimental in approach, as assignment to condition was determined by existing subject characteristics, and no intervention was applied.

Recruitment of patients was performed using a stratified sampling approach such that age, gender, and primary diagnosis (BD, MDD, or SZ) would be similar for TD and non-TD groups. Patients were recruited for the study from a variety of sources, including pre-existing participant databases, patient advocacy groups, and clinicians.

### Patient selection

Participants were recruited by MedQuest Global Marketing Research, a research firm that identifies patients that meet pre-determined study inclusion criteria. MedQuest utilized e-mail lists provided by patient advocacy/support groups, and relationships with doctors/clinicians to recruit and enroll participants.

MedQuest asked that interested groups or clinics contact patients about the study. Potential participants contacted MedQuest, through which arrangements were made to complete an informed consent form (ICF), Authorization to Use and Disclose Protected Health Information for Research, patient screening form, and clinician screening form. The patient screening forms included items regarding psychiatric diagnoses, TD status, and demographic questions. The clinician screening form included items about patients’ psychiatric diagnoses and severity, TD status and severity (if applicable), and medical exclusion criteria (described below). Patients were instructed to bring the form to their current treater, who completed the form especially for participation in this study. If the participant met all of the eligibility criteria (described below), they were e-mailed a link to the survey.

Patients were eligible for inclusion if they had a clinician-confirmed diagnosis of BD, MDD, or SZ, were at least 18 years of age, were willing and able to provide informed consent, and were able to complete the survey online and in English. Patients were excluded from participation in the survey if they had a clinician-confirmed history of traumatic brain injury, debilitating stroke, or the presence of neurodegenerative disease (i.e. Parkinson’s disease, Alzheimer’s disease, or amyotrophic lateral sclerosis), as these conditions may include symptoms that are similar to TD, or if they were currently pregnant or had been pregnant in the previous 6 months, as there was concern regarding the potential for changes in patient medication regimens.

Upon completion of the survey, patients received a $75 honorarium in exchange for their time and effort. The survey opened on May 11, 2017 and closed on December 12, 2017 after the recruitment goal of 450 subjects was reached. All participants resided in the United States. This study was approved by an IRB and all participants gave consent prior to participation.

### Assessment of TD

TD status and severity were captured by two items on the clinician screening form. The first asked the clinician: “Does the patient currently have tardive dyskinesia (TD)?” and had response options of “yes” or “no.” The second, answered only for patients who had been diagnosed with TD, inquired as to the status of the patient’s TD, and read: “Please rate the severity of the patient’s TD (check only one)” and had response options of “mild,” “moderate,” or “severe.”

### Survey measures for HRQoL and social withdrawal

The survey was constructed after a review of literature describing instruments used to measure HRQoL and social withdrawal in patients with BD, MDD, and SZ. An evaluation of the psychometric properties of each of the identified instruments informed the ultimate selection of instruments for the study. The set of instruments comprising the final survey included a generic instrument, the SF-12v2^®^ Health Survey (SF-12v2) [18]; a psychiatric-specific measure of HRQoL, the Quality of Life Enjoyment and Satisfaction Questionnaire, Short Form (Q-LES-Q-SF) [19]; and a measure of social withdrawal due to self-perceived stigma, the Social Withdrawal subscale of the Internalized Stigma of Mental Illness Scale (SW-ISMI) [20].

#### HRQoL: SF-12v2 Health Survey (SF-12v2)

The SF-12v2, a 12-item, self-reported general health questionnaire, was used to measure functioning and well-being. The psychometric properties, including reliability, of the SF12v2 are well established across numerous health conditions and diseases, including psychiatric populations [18]. The SF-12v2 assesses eight health domains: physical function (PF), role limitations due to physical problems (RP), bodily pain (BP), general health (GH), vitality (VT), social function (SF), role limitations due to emotional problems (RE), and mental health (MH). Scores are summarized in two overall health components: the physical health component (PCS, mainly reflecting the PF, RP, BP, and GH domains), and the mental health component (MCS, mainly reflecting the MH, RE, SF, and VT domains). All scores, including MCS and PCS, have a mean of 50 and a standard deviation of 10 in the general population of US adults, with a higher score indicating better functioning and well-being [21].

The SF-12v2 uses two items to assess PF. However, considering the physical manifestations of TD, the PF domain was assessed using a longer scale, the 10 PF items from the SF-36v2 survey, the parent survey of the SF-12v2 [22].

#### HRQoL: Quality of Life Enjoyment and Satisfaction Questionnaire Short Form (Q-LES-Q-SF)

The Q-LES-Q-SF, a 16-item survey, has proven to be suitable for assessing HRQoL in psychiatric populations [19]. The Q-LES-Q-SF has been shown to have good psychometric properties, including good internal consistency and test–retest reliability [19]. The first 14 items of the Q-LES-Q-SF contribute a single overall HRQoL score that ranges from 0 to 1, with scores closer to 1 indicating better HRQoL. The last two of the 16 items, measuring satisfaction with medication and overall life satisfaction, respectively, are stand-alone items not included in the summary score.

#### Social Withdrawal subscale of the Internalized Stigma of Mental Illness scale (SW-ISMI)

The SW-ISMI, a six-item short form of the 29-item ISMI survey, was used to measure exacerbation of mental illness stigma on social functioning due to TD [20]. The full survey comprises five subscales: Alienation, stereotype endorsement, discrimination experience, social withdrawal, and stigma resistance; however, participants in the current study completed only the social withdrawal scale, which has shown good internal consistency and test–retest reliability [20].The final score, which can have a maximum value of 4 for severe social withdrawal, is the sum of all values from each item divided by the total number of answered items.

### Outcomes

#### Group differences by TD status and diagnosis

One-way analyses of covariance (ANCOVAs), which included covariates (age and gender) to account for variation due to demographic variables, were performed for each domain to compare SF-12v2, Q-LES-Q, and SF SW-ISMI scores between patients with and without TD. This type of ANCOVA was conducted for the pooled group of patients with BD, MDD and SZ, and for each diagnosis type alone.

#### Group differences by clinician-rated TD severity and diagnosis

When clinicians rated patients’ TD severity on a 3-point scale (mild, moderate, or severe), very few patients were rated as having severe TD (4.1%). Thus, patients who were rated as having moderate or severe TD were collapsed to create one group, described as moderate/severe TD. Subsequently, one-way ANCOVAs, which included demographic covariates (age and gender), were performed for each domain to compare SF-12v2, Q-LES-Q, and SF SW-ISMI scores between patients without TD, mild TD, or moderate/severe TD. This type of ANCOVA was conducted for the pooled group of patients with BD, MDD, and SZ, and for each diagnosis type alone.

#### Burden of disease analyses

To evaluate the health status burden associated with TD, one-way ANOVAs were performed to compare the baseline SF-12v2 scores from the study sample with benchmark scale scores from the US general population. To evaluate the health status burden associated with TD, one-way ANCOVAs were performed to compare the baseline SF-12v2 scores from the study sample with normative sample scale scores from the US general population. To control for differences in key sample characteristics between the current and normative samples, the normative sample was adjusted to match the age and gender distribution of the current sample or subsample (i.e., for the total sample and separately for each condition) using separate least squares multiple regression models for each of the SF-12v2 scales and component summaries. Weights for mean scores for each benchmark sample were then estimated based on these matched sample characteristic demographics, with mean scores and standard errors then adjusted based on these weights.

### Statistical analyses

Significant differences for all comparisons were assessed at an alpha level of 0.05 with no correction for multiplicity, as these were exploratory analyses. The data are presented as group mean ± standard error. The interpretation of differences between groups in the SF-12v2 survey were based on recommended values for the minimal important difference (MID). These differences have been derived based on analyses of important health consequences such as risk of mortality and hospitalization, as well as studies of score differences for groups known to differ on physical and mental health [22]. In addition, Cohen’s *d* effect size (ES) [23] for standardized differences was calculated and is presented as an absolute value to interpret the magnitude of difference in HRQoL between the patients in this study and the general population. Criteria for defining the level of clinical meaningfulness were as follows: ES < 0.2 = negligible; 0.2 ≤ ES < 0.5 = small; 0.5 ≤ ES < 0.8 = moderate and ES ≥ 0.8 = large.

To address concerns that the distributions of dependent variables may have violated the assumptions of statistical tests, analyses were performed wherein the continuous dependent variables were recoded into five equal-sized categories (low, low middle, middle, high middle, high) and submitted to ordinal logistic regressions with TD status as the predictor. These tests are not influenced by outliers, as the distance between scores is removed when recoded.

## Results

### Study population

Among 459 patients who completed the online survey, 416 were included in the study. A total of 43 participants were excluded for the following reasons: four did not meet the eligibility requirements, one requested to be withdrawn from the study, one had someone else fill out the survey, three had survey responses that could not be accurately linked to diagnostic information provided at screening, and 34 did not provide consistent answers based on checks of the logical consistency of eight pairs of responses to individual survey items (e.g., indicated that they were “limited a lot” in walking 100 yards, but were “not limited at all” in walking a mile, etc.). Of the study population, 219 did not have TD, while 197 had TD. Of those diagnosed with TD, clinician assessments indicated that 109 had mild TD (55.3%) and 88 had moderate/severe TD (44.7%). The average time to complete the survey was 8.5 min (IQR = 4.73–10.76).

Demographic and clinical characteristics of the survey population as well as the patient-reported outcome scale distributions are summarized in Table 1. The mean age was similar between the non-TD and TD groups, and both groups had a similar proportion of male and female patients. Each of the diagnoses (BD, MDD, SZ) made up approximately one-third of the non-TD and TD groups.

**Table 1:** Patient demographic and clinical characteristics

	Non-TD (*n *= 219)	TD (*n *= 197)	All (*N *= 416)
**Age, mean (SD)**	43.93 (13.3)	44.39 (13.1)	–
**Female, *n*(%)**	108 (49.3)	95 (48.2)	203 (48.8)
Diagnoses, *n*(%)*			
Schizophrenia	73 (33.3)	60 (30.4)	133 (32.0)
Bipolar disorder	74 (33.8)	69 (35.0)	143 (34.4)
Major depressive disorder	72 (32.9)	68 (34.5)	140 (33.7)
Clinician-rated TD severity, *n*(%)*			
Mild	–	109 (55.3)	–
Moderate	–	80 (40.6)	–
Severe	–	8 (4.1)	–
**PCS**			
Mean (SD)	48.76 (10.3)	44.58 (10.76)	46.77 (10.71)
Q25/Q50/Q75	41.91/49.73/56.9	36.26/45.14/52.66	39.89/47.83/55.26
**MCS**			
Mean (SD)	38.44 (10.71)	36.36 (10.15)	37.45 (10.49)
Q25/Q50/Q75	30.53/38.18/45.09	30.17/35.92/43.1	30.21/36.93/44.65
**PF**			
Mean (SD)	47.37 (10.56)	42.93 (11.42)	45.26 (11.19)
Q25/Q50/Q75	40.32/49.88/55.63	34.57/44.15/53.71	36.49/47.97/55.63
**Q-LES-Q-SF**			
Mean (SD)	0.53 (0.21)	0.46 (0.2)	0.5 (0.21)
Q25/Q50/Q75	0.39/0.52/0.68	0.32/0.45/0.61	0.36/0.5/0.66
**SW-ISMI**			
Mean (SD)	2.3 (0.74)	2.52 (0.82)	2.4 (0.79)
Q25/Q50/Q75	1.83/2.33/2.83	2/2.67/3	2/2.5/3

*MCS* mental component summary of the SF-12v2, *PCS* physical component summary of the SF-12v2, *PF* physical functioning of the SF-36v2, *Q25* first quartile, *Q50* median, *Q75* third quartile, *Q*-*LES*-*Q*-*SF* Quality of Life Enjoyment and Satisfaction Questionnaire Short Form, *SD* standard deviation, *SW*-*ISMI* Social Withdrawal subscale of the Internalized Stigma of Mental Illness scale, *TD* tardive dyskinesia

*Totals adding up to less than 100% are based on rounding to one decimal place

### Effects of TD status on HRQoL

**Fig. 1:**
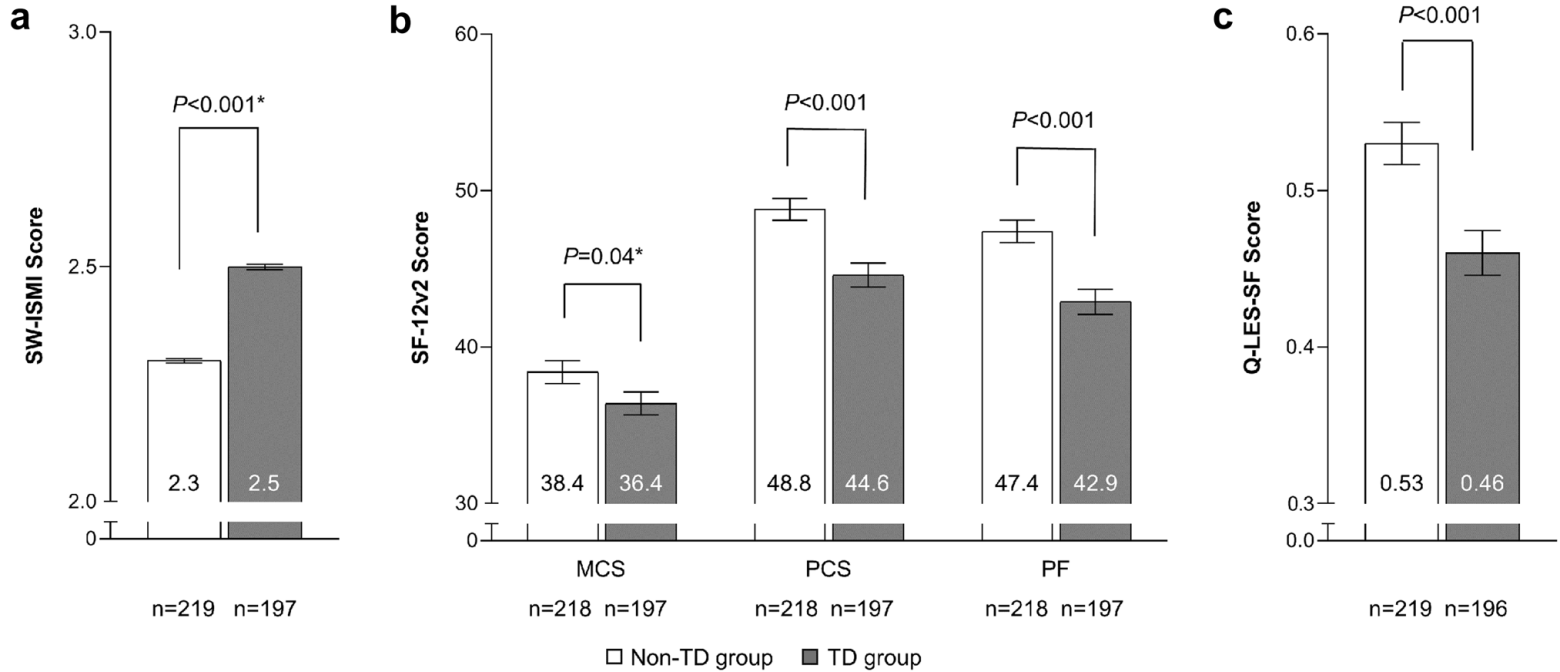
Health-related quality of life in patients with and without TD. **a** SW-ISMI score for patients without TD vs those with TD. For the SW-ISMI scale, higher values indicate worse social withdrawal. **b** SF-12v2 score and **c** Q-LES-SF score for patients without TD vs those with TD. For the SF-12v and Q-LES-Q SF scales, higher scores indicate better quality of life status [21]. *Not significant when model includes severity of BD, MDD, or SZ as measured by CGI on a seven-point scale. *BD* bipolar disorder, *CGI* clinical global impression, *SW-ISMI* Social Withdrawal subscale of the Internalized Stigma of Mental Illness scale, *MCS* mental component summary of the SF-12, *MDD* major depressive disorder, *PCS* physical component summary of the SF-12, *PF* physical functioning of the SF-36v2, *Q-LES-Q SF* Quality of Life Enjoyment and Satisfaction Questionnaire Short Form, *SF-12* SF-12v2 Health Survey, *SZ* schizophrenia, *TD* tardive dyskinesia. The data show group mean ± standard error, represented by the error bars

In the pooled group of patients with BD, MDD, or SZ, HRQoL (SF-12v2 and Q-LES-SF) and social withdrawal (SW-ISMI) were compared between patients with and without TD (Fig. 1). Patients without TD diagnosis had significantly better HRQoL and less social withdrawal than patients with TD (SW-ISMI score: 2.3 vs. 2.5; SF-12v2 MCS score: 38.4 vs. 36.4; SF-12v2 PCS score: 48.8 vs. 44.6; SF-12v2 PF score: 47.4 vs. 42.9; Q-LES-SF score: 0.53 vs. 0.46; all *P *< 0.001, except for MCS, *P *= 0.04). The differences exceeded the 2-point MID for PCS (score difference = 4.18) and the 3-point MID for PF (score difference = 4.44) but did not exceed the 3-point MID for MCS (score difference = 2.08).

Similar results were obtained when examining BD, MDD, and SZ separately. Compared with patients without TD, patients with TD had worse HRQoL and more social withdrawal. MID thresholds were exceeded for PCS, MCS, and PF score differences in the SZ group but not in the BD and MDD groups. The MID was exceeded only in the PCS domain for patients with BD (score difference = 2.48) or MDD (score difference = 3.01). The score differences in all domains were larger for the SZ group than for the BD and MDD groups, as patients without TD achieved the best scores and patients with TD had the worst scores of all.

### Association between clinician-rated TD severity and HRQoL

**Fig. 2:**
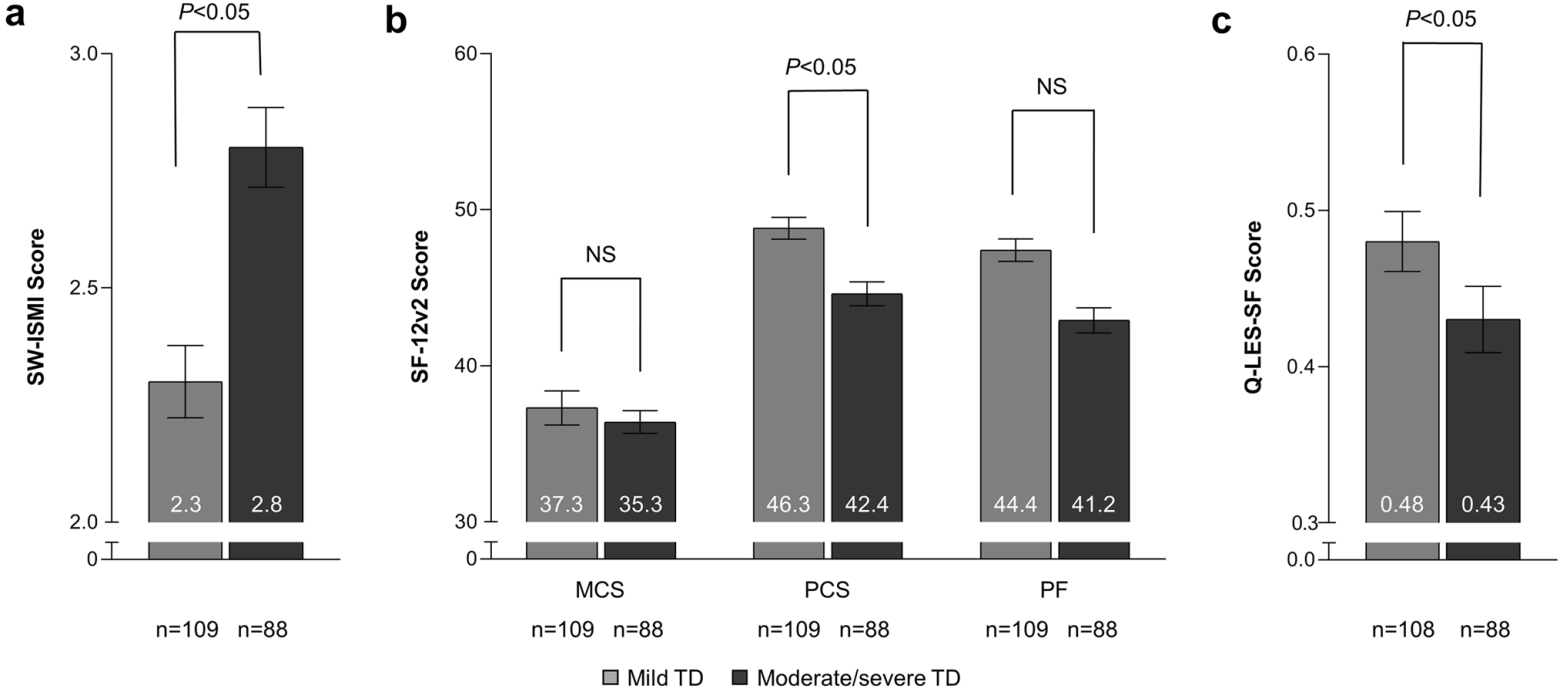
Health-related quality of life in TD patients stratified by clinician-reported symptom severity. **a** SW-ISMI score stratified by clinicians for patients with mild vs moderate/severe TD. For the SW-ISMI scale, higher values indicate worse social withdrawal. **b** SF-12v2 score and **c** Q-LES-SF score stratified by clinicians for patients with mild vs moderate/severe TD. For the SF-12v and Q-LES-Q SF scales, higher scores indicate better quality of life status [21]. *MCS* mental component summary of the SF-12, *PCS* physical component summary of the SF-12, *NS* not significant, *Q-LES-Q SF* Quality of Life Enjoyment and Satisfaction Questionnaire Short Form, *PF* physical functioning of the SF-36v2, *SF-12* SF-12v2 Health Survey, *SW-ISMI* Social Withdrawal subscale of the Internalized Stigma of Mental Illness scale, *TD* tardive dyskinesia. The data show group mean ± standard error, represented by the error bars

The impact of different levels of TD severity on patients’ HRQoL and social withdrawal was examined in the pooled group of patients with BD, MDD, and SZ by comparing the outcomes of patients that had clinician-rated mild TD with those that had clinician-rated moderate/severe TD. Patients with mild TD scored better on all scales than did patients with moderate/severe TD (Fig. 2), and these differences were significant (*P *< 0.05) for the following scales; SW-ISMI: 2.3 versus 2.8; SF-12v2-PCS: 46.3 versus 42.4; Q-LES-Q-SF: 0.48 versus 0.43. Scores that were better for patients with mild TD but that were not significantly different from patients with moderate/severe TD were SF-12v2-MCS: 37.3 versus 35.3 and SF-12v2-PF: 44.4 versus 41.2.

### Disease burden

**Fig. 3:**
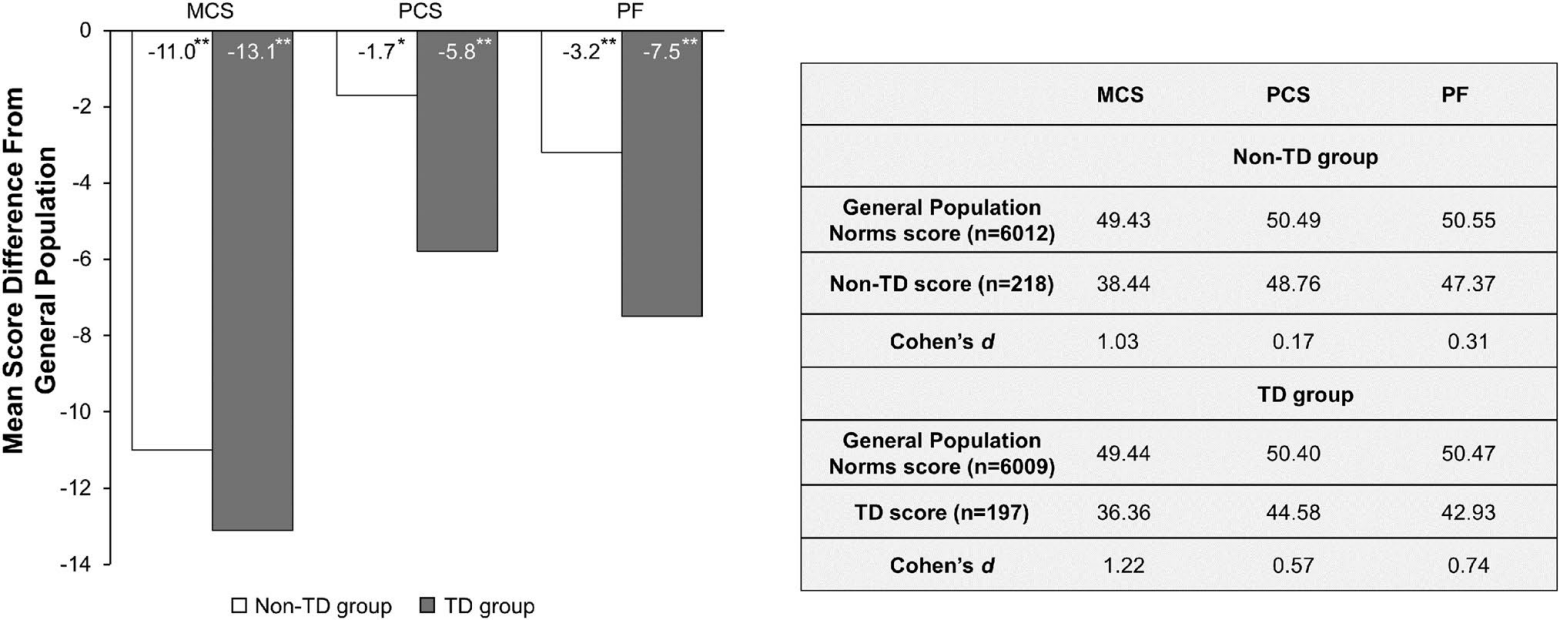
MCS, PCS and PF Scores: difference from the general population in patients with and without TD. For MCS, PCS, and PF, higher scores indicate better health status [21]. The difference from the general population was weighted to the gender and age of the patient samples. *P = 0.015 (comparing the mean value with the general population mean); **P < 0.001 (comparing the mean value with the general population mean). *MCS* mental component summary of the SF-12v2, *PCS* physical component summary of the SF-12v2, *PF* physical functioning of the SF-36v2, *TD* tardive dyskinesia

To evaluate the health status burden associated with TD, baseline SF-12v2 (MCS, PCS, and PF) scores from the pooled group with BD, MDD, and SZ were compared with adjusted benchmark scores from the US general population (Fig. 3). In the non-TD group patients had significantly lower scores in all three domains of the SF-12v2 than the general population norm. The largest difference was observed in the MCS domain (− 11.0; Cohen’s *d *= 1.03), followed by the PF domain (− 3.2; Cohen’s *d *= 0.31), and the PCS domain (− 1.7; Cohen’s *d *= 0.17).

Notably, presence of TD further increased the score differences between the pooled group of patients (BD, MDD, and SZ) and the general population norm in all three domains of the SF-12v2. The largest difference was again observed in the MCS domain (− 13.1; Cohen’s *d *= 1.22), followed by the PF domain (− 7.5; Cohen’s *d *= 0.74); and PCS domain (− 5.8; Cohen’s *d *= 0.57).

Each mental illness alone (BD, MDD, or SZ) had at least a moderate impact on the score of the MCS domain (BD: Cohen’s *d *= 1.12; MDD: Cohen’s *d *= 1.19; SZ: Cohen’s *d *= 0.74) but only a small or negligible effect (Cohen’s *d *< 0.5) on the PF and PCS scores (Table 2). Presence of TD increased the burden observed with each mental illness in the SF-12v2 domains at least to a moderate level (Cohen’s *d *≥ 0.5), with exception of the PCS domain, in which the impact of TD remained small in patients with BD (Cohen’s *d *= 0.43) or MDD (Cohen’s *d *= 0.48). Patients with SZ were most sensitive to TD compared with those with BD or MDD, resulting in a large impact of TD on all three SF-12v2 domains (Cohen’s *d *> 0.8).

**Table 2:** SF-12v2 score differences from the age- and gender-adjusted general population norm

Mean difference from general population norm sample	MCS	PCS	PF
BD sample (*n *= 74)	− 11.7**	− 1.96	− 3.59*
BD + TD sample (*n *= 69)	− 12.72**	− 4.43**	− 6.32**
MDD sample (*n *= 72)	− 13.41**	− 1.98	− 4.07*
MDD + TD sample (*n *= 68)	− 13.42**	− 4.98**	− 6.23**
SZ sample (*n *= 73)	− 7.91**	− 1.25	− 1.89
SZ + TD sample (*n *= 60)	− 13.11**	− 8.39**	− 10.42**

*BD* bipolar disorder, *MCS* mental component summary of the SF-12v2, *MDD* major depressive disorder, *PCS* physical component summary of the SF-12v2, *PF* physical functioning of the SF-36v2, *TD* tardive dyskinesia, *SZ* schizophrenia

*P < 0.05 (comparing the mean value with the general population mean); **P < 0.001 (comparing the mean value with the general population mean)

These results show that the burden associated with mental health (MCS score) was high due to the underlying mental illness (BD, MDD, or SZ), while only a minor effect was observed on physical health (PF and PCS scores). Presence of TD had an additional impact on all three SF-12v2 domains with more dramatic effects on the scores associated with physical health.

Sensitivity analysis using ordinal logistic regression supported the results obtained from the primary analyses by showing that TD status was a significant predictor of all dependent measures (PF: *P *< 0.001, PCS: *P *< 0.001, SW-ISMI: *P *= 0.012, Q-LES-Q-SF: *P *= 0.001) except MCS (*P *= 0.095).

## Discussion

This study investigated the impact of TD on HRQoL and social withdrawal using a survey that contained items from the SF-12v2, Q-LES-Q-SF, and SW-ISMI questionnaires, and which was completed by patients with different underlying mental illnesses (BD, MDD, or SZ). To date and to the best of our knowledge, different aspects of HRQoL in patients with TD, such as mental and physical health, have not been addressed elsewhere.

In the pooled group combining BD, MDD, and SZ, patients with TD had significantly worse HRQoL and increased social withdrawal than patients without TD and the general population norm. Notably, the burden of TD increased with escalating severity of the condition. These results are consistent with previous studies that investigated the effect of TD on quality of life in patients with SZ [17, 24]. However, although these reports clearly demonstrate a negative impact of TD on patients with SZ, the impact of TD on other populations was not investigated.

In this study, results from the SF-12v2 questionnaire show that physical health-related differences between the TD and non-TD groups were much larger than differences related to mental health. Similarly, the physical health burden in patients with TD compared with the general population norm was higher than in patients without TD, while mental health burden was high in patients with BD, MDD, or SZ regardless of TD status. In addition, decreases in HRQoL with increasing TD severity were mostly due to decreased physical health. These observations indicate that presence of TD has a substantial impact on physical health burden in patients who already experience a significant mental health burden due to the underlying mental illness.

Considering the nature of the SF-12v2 physical functioning items (climbing stairs, walking, carrying groceries, etc.), which mostly map onto activities requiring gross motor movements, it is difficult to reconcile how small involuntary movements of the face and extremities can predict differences not only by the presence or absence of TD, but also by TD severity. While any posited mechanism is speculative, one possibility is that those suffering from TD lose some level of confidence in their physical abilities as a result of the involuntary movements they experience. This would not necessarily mean that changes in physical functioning are simply a change in perception. In fact, we see it as more likely that this lack of confidence would influence physical functioning, and we suggest that the SF-12v2 physical functioning items are reflecting real changes that result from this lack of confidence.

Interestingly, when analyzing each mental illness separately, the SZ group was more sensitive to TD than the BD and MDD groups. In the absence of TD, patients with SZ had a comparable physical health status with the general population norm and a mental health status that was moderately affected. Surprisingly, both of these scores were better in the SZ group in the absence of TD than those observed in the BD and MDD groups in the absence of TD. Although we cannot rule out the possibility of sampling error, this unexpected result could be a consequence of the progressive nature of SZ, which may result in the gradual acceptance of one’s condition over time [25] versus the more-episodic appearance of BD and MDD. However, when patients also had TD in addition to SZ, both, mental- and physical health-related scores were significantly lower. Moreover, patients with SZ and TD had the lowest score on all scales compared with the respective BD or MDD groups.

Thus, in the pooled group of patients with BD, MDD, and SZ observations regarding differences on all scales between patients with and without TD were driven by those who had SZ. However, the burden of TD in patients with BD or MDD should not be discounted, since having TD was associated with overall worse HRQoL, in particular physical health, for both conditions.

This study has implications for clinicians prescribing antipsychotic medication, as it clearly demonstrates that TD is not simply a nuisance side effect. Rather, TD impacts patients’ physical well-being and exacerbates existing issues of stigma related to their underlying condition. Considering that treatment with antipsychotics is one of the only options available for some patients, clinicians might look for treatments for TD that can be administered in conjunction with antipsychotic medications. Future work might investigate the mechanisms by which physical functioning is impacted by TD, to better characterize how these involuntary movement of the extremities and face influence the larger scale type of physical functioning captures by the SF-12v2. The impact of TD on antipsychotic medication adherence is also an important area for mental health treaters to understand. Ultimately, methods of treatment for TD need to be evaluated in light of patient experience, including HRQoL and stigma.

## Study limitations

This study has several limitations. Observations presented here may be directly associated with TD but may also be influenced by other pre-existing differences, such as comorbidities, that are beyond the control of this study. Therefore, this study accounted for additional factors such as age and gender that could potentially act as a confound for TD. However, it is worth noting that severity of a mental illness may in fact be an important consideration in assessing TD status, as patients with severe mental illness more likely have a greater exposure to antipsychotic medication that causes TD. Statistical tests did not control for multiple comparisons. However, most tests of differences had *P* values of < 0.001 and would be significant even with adjustment for multiple comparisons. While sensitivity analyses using ordinal logistic regression showed TD status to be a significant predictor of PCS, PF, ISMI and Q-LES-Q, MCS was not quite statistically significant (*P *= 0.095) using this lower-powered test. Out of concern that we could not assume that clinicians’ ratings of mental illness severity and TD severity were independent of each other, we did not include severity of mental illness as a covariate in analyses. Another caveat of the study design was that the clinicians’ ratings of TD severity were not validated with a more established method, such as AIMS.

## Conclusions

Although patients with BD, MDD, or SZ experience a deficit in HRQoL and notable social withdrawal, the presence of TD further impacts their health status, with the strongest impact on physical health. The key concepts related to HRQoL in patients with TD identified here may be assessed in future clinical trials.
